# The Impact of Streetlight Placement on Trends and Disparities in Pedestrian-Motor Vehicle Crashes

**DOI:** 10.1007/s11524-026-01098-8

**Published:** 2026-06-30

**Authors:** Cassandra Peitzman, Kanglin Chen, Kori S Zachrison, Margaret Samuels-Kalow

**Affiliations:** 1 Department of Emergency Medicine, Brigham and Women’s Hospital, Harvard Medical School, 10 Vining Street, Neville House, Boston, MA 02115 USA; 2 Government Department, Faculty of Arts and Sciences, Center for Geographic Analysis, Harvard University, Harvard CGIS South Building, 1737 Cambridge St., Cambridge, MA USA; 3 Department of Emergency Medicine, Massachusetts General Hospital, Harvard Medical School, 125 Nashua St, Boston, MA 02114 USA

**Keywords:** Motor vehicle crash, Pedestrian, Streetlight, Trauma, Road safety, Injury prevention, Accidental Injury

## Abstract

Pedestrian-motor vehicle crashes are a major cause of preventable injury in urban environments. They have increased markedly over the past several decades, especially overnight, and with a disproportionate impact on pedestrians of color. Streetlights represent a potential tool to combat these trends, but empirical studies of their relationship with pedestrian trauma are limited. We use geospatial data from Boston (2016–2023) to assess the relationship between streetlight density (lights/road kilometer) by census tract on (1) pedestrian crashes, (2) changes in crash rates over time, (3) racial disparities in crash incidence, and (4) proportion of crashes occurring overnight. Pedestrian crashes decreased from 2016 to 2023 from 616 (0.55/km) to 421 (0.37/km), with higher streetlight densities associated with greater overall reductions (an additional −0.002 crashes/km/year per streetlight/km (95% CI [−0.003, −0.001]). Persistent racial disparities were observed, with 0.04 additional crashes/km/year (95% CI [0.02, 0.06]) per 10% residents of color. However, this association decreased by 0.004 crashes/km/year (95% CI [−0.01, −0.001]), and higher streetlight densities were associated with greater reductions (additional annual decrease of 0.0004 crashes/km/year for every streetlight/km (95% CI [−0.001, −0.0001]). Finally, the proportion of crashes occurring overnight increased from 37.6% to 45.1%. Streetlight density was negatively associated with overnight timing (adjusted odds ratio of 0.991; 95% CI [0.986, 0.997] per streetlight/km) but had no relationship with the time trend. We conclude that there may be a beneficial relationship between streetlight density and pedestrian safety, where census tracts with higher streetlight densities demonstrated greater improvements in overall crash rates and racial disparities.

**Supplementary Information:**

The online version contains supplementary material available at https://doi.org/10.1007/s11524-026-01098-8.

## Introduction

Pedestrian-motor vehicle crashes are an important cause of morbidity and mortality in the United States. They account for over 60,000 injuries and 7000 deaths annually, and crash rates have increased dramatically since 2009, especially at night [1, 2]. The cause of this increase is likely multifactorial, with potential contributors including population movement toward areas with less developed pedestrian safety infrastructure, expansion of suburban residential areas closer to large roadways (especially for lower income populations), and increases in “distracted driving” [3, 4].

Unfortunately, in addition to increasing overall, pedestrian injuries have increased disproportionately for Black, Hispanic, and Multiracial pedestrians [3, 5–8]. In Boston, for example, Black and Hispanic pedestrians are approximately four times as likely to be struck by a vehicle than their white counterparts [8]. Inequities in safety infrastructure may contribute to these differences, with previous work finding links between pedestrian trauma and historical segregation and infrastructure investment [6].

Multiple elements of transportation safety infrastructure, including streetlights, sidewalks, and traffic-calming measures in high-risk areas, have been shown to reduce pedestrian injuries in small-scale studies [9–13]. Previous studies using police records have also found an association between the presence of street lighting and decreased fatality (though not frequency) of nighttime crashes [2]. However, detailed analysis of the relationship between streetlight density and pedestrian crashes has been limited by data availability.

The recent publication of state- and city-level geospatial data through projects such as the “Vision Zero” initiative have made larger, more detailed assessments of these relationships possible. This paper uses geospatial roadway, streetlight, and emergency medical services (EMS) data available through Boston’s “Analyze Boston” program to examine the relationship between roadway streetlight density and (1) pedestrian crash rates, (2) changes in crash rates over time, (3) the association between these trends and census-tract racial composition, and (4) the proportion of crashes occurring overnight for the city of Boston from 2016 to 2023.

## Methods

### Data

Data were taken from multiple public datasets. Boston Vision Zero Crash Records provided the date, time, and location of all pedestrian-involved accidents requiring Emergency Medical Services (EMS) activation within Boston from January 1, 2015, to December 31, 2023. (Of note, primary analyses were restricted to 2016–2023 for overlap with streetlight data and to avoid bias related to methodological changes after 2023 [14]). Streetlight geolocations were obtained from the 2016 Boston Streetlight Inventory Dataset and the 2022 City of Boston Streetlight Audit Dashboard [15, 16]. Census tract outlines and 2010 demographic data were taken from the Boston Climate-Ready Social Vulnerability Index shapefile [17]. Spatial data for Boston-managed streets, as well as average annual daily traffic (AADT) by road segment from the 2017 Boston Roads Inventory, were obtained from the City of Boston Managed Streets 2020 shapefile [18]. Geographic zoning data (downtown vs neighborhood) were taken from the 2025 Boston zoning code dataset and shapefile, supplemented by the 2013 *Boston Complete Streets Design Guidelines* [19, 20]. Finally, census-tract demographic data (including resident race and population) were provided by the 2020 American Community Survey, with standardization to 2010 census-tract boundaries by the National Historical Geographic Information System [21].

#### Data Processing

Analyses included eight full years of crash records (January 1, 2015, to December 31, 2023) across residential census tracts within the city of Boston.

Spatial processing and analyses were performed in ArcGIS Pro (3.1.0). All census tract data were mapped to 2010 census tract boundaries. Census tracts were chosen as the primary level of analysis for concordance with census demographic data. Pedestrian crashes and streetlights located within 15 m of a Boston-managed street were included in our analysis. This threshold was found to include roadside streetlights without capturing streetlights present on parallel roads or in nearby parks, parking lots, or other non-roadway areas which tended to be physically obscured from roadways. Special and non-residential census tracts (e.g., parks) with fewer than 10 housing units or 100 residents were excluded from analysis.

Variables were calculated at the census-tract level using the above sources and included yearly pedestrian crashes per road kilometer, streetlights per road kilometer (for both 2016 and 2022 inventories), and majority zoning category (downtown vs neighborhood) to account for differences in housing density, roadway and pedestrian infrastructure regulations, average annual daily traffic (1000 vehicles/day), population density (1000 residents/km^2^), and percent residents of color (percent of residents not identifying as White-Non-Hispanic). An additional variable determining whether a crash occurred overnight (between sunset and sunrise) was created by comparing EMS dispatch time to date-matched Boston sunrise and sunset times, calculated with the R “suncalc” package [22].

### Analysis

Four primary analyses were performed using multivariable regression.

*Streetlight Distribution*: Our first analysis assessed census-tract characteristics associated with streetlight density in the 2016 and 2022 inventories, including pedestrian crashes in preceding years, average daily traffic volume (1000 vehicles/day in the 2016 inventory), zoning category (majority downtown vs neighborhood), and population density (residents/km^2^). Additional analysis assessed the relationship between census-tract percent residents of color (people of color as a percentage of total census tract population) and streetlight density (streetlights/km of Boston-managed roadway). Of note, crash records from 2015 were used as a lagged variable in the analysis of 2016 streetlight allocation but excluded from primary crash and time-trend analyses given the absence of same-year streetlight data.

*Overall* *Pedestrian* *Crashes*: We then assessed the relationship between total census-tract pedestrian crash rates (crashes/km/year) and (1) streetlight density and (2) census tract percent residents of color across all years. Controls were included for average daily traffic volume, zoning category, and population density. We also assessed for moderation of the relationship between percent residents of color and crash density (racial disparity in crash incidence) by streetlight density.

*Time* *Trends*: Third, we assessed the relationship between time trends in pedestrian crash rates (in crashes/km/year) and each of the above variables (average daily traffic, zoning category, population density, and census tract percent residents of color) as well as streetlight density. Interaction terms were included to assess for time-related changes in racial disparities and to assess for changes in the moderation of disparities by streetlight density.

*Overnight* *Occurrence*: Finally, logistic regression models were used at the individual crash level to examine both time trends in overnight (sunset to sunrise) crash occurrence and the impact of streetlight density on the probability of a given crash occurring at night. Given the potential for volatility in crash timing associated with differential transit patterns during the 2020–2021 COVID-19 pandemic, fixed effects for these years were also included in time trend analyses [23].

*Modeling* C*onsiderations:* All models of crash density by census tract (crashes/km) were weighted for total census-tract road kilometers to generate city-wide estimates. Primary time-trend analyses were performed using data from the 2022 Boston streetlight audit, with sensitivity analysis using the 2016 streetlight inventory. Given methodological differences between the two streetlight surveys, they were considered individually.

Heteroskedasticity- and autocorrelation-robust standard errors were used for all analyses, with clustering at the census-tract level. Spatial autocorrelation of crashes and streetlight densities was confirmed using a Global Moran’s I statistic in ArcGIS Pro (3.1.0). All other statistical analyses were performed in R (4.3.3) [24].

## Results

Approximately 1320 km of roadway were analyzed across 169 census tracts with an average of 43 streetlights/km. Over the 8 years (2016–2023) of our primary analysis, there were an average of 566 annual crashes (0.43/km), or a total of 4529 pedestrian crashes (Table 1).

**Table 1:** Summary statistics including road length (km), geographic area (km^2^), streetlight count, average daily traffic volume, population and percent residents of color, both overall and by census tract, as well as the distribution of total and overnight pedestrian crashes by year

Census tract characteristics					Neighborhood zoning			Downtown zoning		Full city	
					Census tract means						
Road length (km)					8.5			5.5		*N*. = 1324.13; mean = 7.8	
Area (km^2^)					0.74			0.39		*N*. = 112; mean = 0.66	
N. streetlights (2016)					253			369		*N*. = 46,983; mean = 278	
Streetlights/km (2016)					30			67		Mean = 35	
N. streetlights (2022)					330			374		*N*. = 57,282; mean = 339	
Streetlights/km (2022)					39			68		Mean = 43	
Average daily traffic (1000 vehicles/day)					5.01			10.02		Mean = 5.77	
Population (1000)					4020			3910		*N*. = 675,476; mean = 3997	
Percent residents of color					59%			33%		Mean = 53%	
N. census tracts					*N*. = 133			*N*. = 36		*N*. = 169	
Annual crashes	Years of Primary Analysis									Reference	
	2016	2017	2018	2019	2020	2021	2022	2023	2016–2023 Totals	2015	2024
N. total crashes	837	726	646	646	376	327	421	550	4529 (503.2/yr)	750	427
Mean crashes/km	0.63	0.55	0.49	0.49	0.28	0.25	0.32	0.42	0.43	0.57	0.32
N. overnight crashes	315	288	250	274	152	121	188	248	1836 (229.5/yr)	276	192
% overnight	0.38%	0.40%	0.39%	0.42%	0.40%	0.37%	0.45%	0.45%	0.41%	0.37%	0.45%

To confirm the presence of spatial clustering among crashes and streetlights, Global Moran’s I test for spatial autocorrelation was performed. This was positive in both cases, with *p* < 0.001 and Global Moran’s I indices of 0.73 and 0.22, respectively, suggesting non-random spatial distribution (clustering) of both variables.

### Streetlight Distribution

Streetlights were more likely to be located in census tracts with higher lagged or preceding year crash rates, with each additional crash/km in 2015 (the first year of crash data availability) associated with an additional 21 streetlights/km in the 2016 inventory (95% CI [15, 24]) and each additional crash/km in 2016–2021 associated with an additional 22 streetlights/km in the 2022 inventory (95% CI [13, 25]), holding other variables constant.

Streetlights were also more densely placed in downtown census tracts and census tracts with higher population density. For the 2016 inventory, there were 23 additional streetlights/km in downtown tracts (95% CI [13, 26]), 2.0 additional streetlights/km per 1000 residents/km^2^ (95% CI [1.5, 2.5]), and 0.96 additional streetlights per 1000 vehicles/day (95% CI [0.30, 1.3]). For the 2022 inventory, there were 22 additional streetlights in downtown census tracts (95% CI [11, 25]) and 1.06 additional streetlights per 1000 residents/km^2^ (95% CI = [0.61, 1.5]) (Table A-1).

#### Racial Disparities

There was no overall relationship between percent residents of color and streetlight density. However, when controls were included for crash rates in the preceding years, streetlight density was lower in census tracts with more residents of color, by an average of 1.7 fewer streetlights/km (95% CI = [−2.8, −0.60]) in the 2016 and 1.6 fewer streetlights (95% CI = [−2.4, −0.9]) in the 2022 inventory per 10% residents of color (Table A-2).

**Fig. 1:**
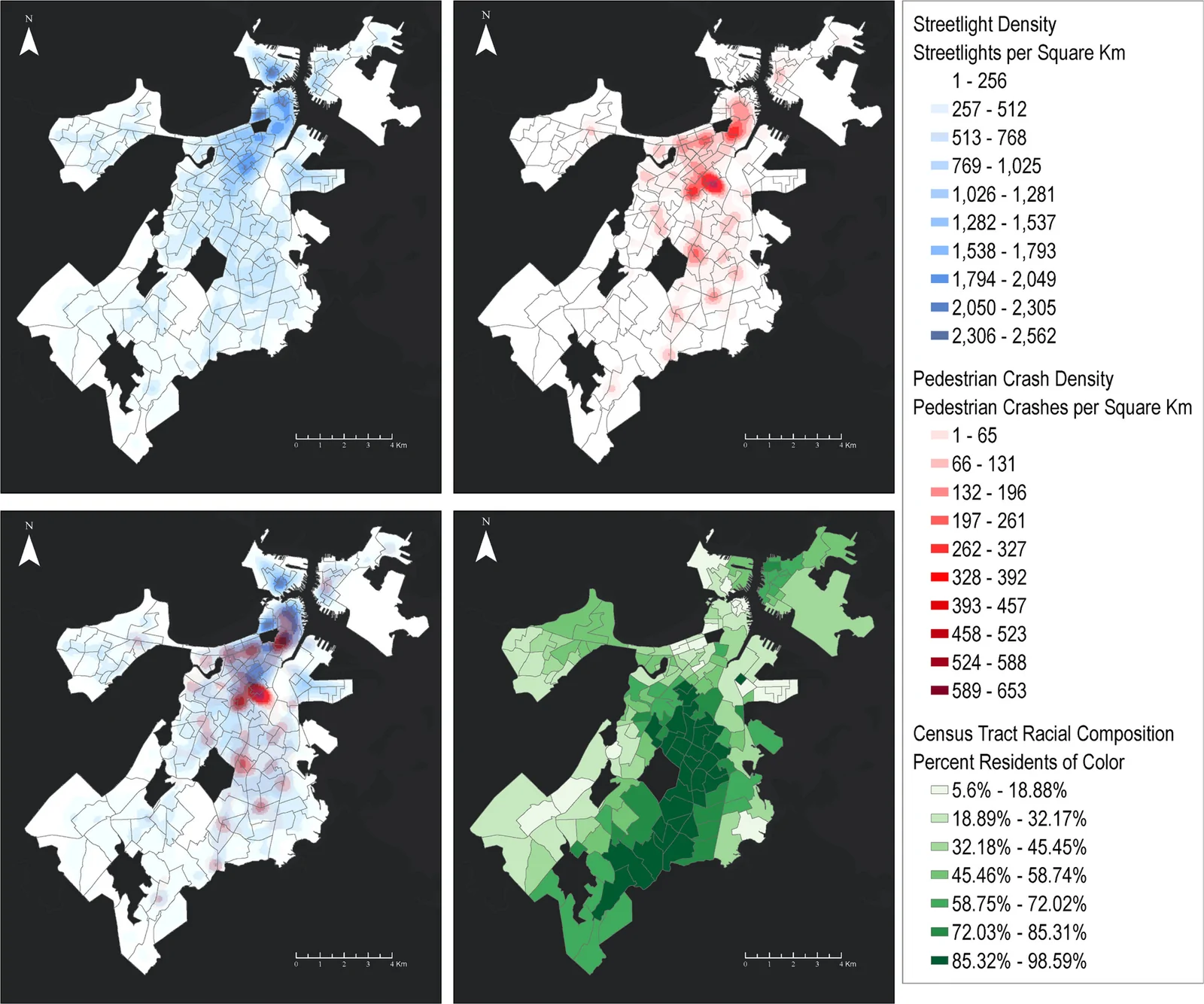
Maps showing **A** streetlight density distribution, **B** density of pedestrian crashes over the study period, **C** the spatial relationship between these distributions, and **D** percent people of color by census tract

This was equivalent to a difference of approximately 8.7 streetlights/km in 2016 (28 vs 37 streetlights/km) and 8.3 streetlights/km in 2022 (39 vs 47 streetlights/km) between the 25th and 75th percentile census tracts (29% vs 80% residents of color). (Figure 1 provides an illustration of the differential overlap between pedestrian crashes and streetlight density by census tract percent residents of color).

### Census Tract Characteristics and Crash Rates

From 2016 to 2023, there were an average of 0.43 pedestrian crashes/km/year on Boston roadways. Within census tracts, downtown zoning, higher population densities and higher daily traffic volumes were associated with higher pedestrian crash rates. Downtown census tracts experienced 0.29 (95% CI = [0.11, 0.48]) additional crashes/km annually compared to neighborhood census tracts, 0.024 additional crashes/km annually for each 1000 additional residents per km^2^ (95% CI [0.012, 0.036]), and 0.034 additional crashes/km for each 1000 vehicles/year/day (95% CI [0.013, 0.055]) (Table A-3).

For a representative census tract with the city mean (7.8) kilometers of roadway, these rates translated to cumulative average of 27.8 pedestrian crashes per census tract from 2016 to 2023, with 18.4 additional pedestrian crashes in “downtown” tracts, 1.5 additional crashes per 1000 residents/km^2^, and 2.12 crashes/1000 vehicles/day.

#### Racial Disparities

Racial disparities in pedestrian crash rates were observed throughout the study period. Overall, each 10% increase in census-tract residents of color was associated with 0.039 additional crashes/km/year (95% CI [0.018, 0.059]), representing a cumulative average difference of 12.4 crashes over the study period (33.2 vs 20.8 total crashes) between an average census tract at the 75th percentile percent (80% residents of color) compared to the 25th percentile (29% residents of color). When controlled for other tract characteristics, there were 0.05 additional crashes/km/year per 10% residents of color (95% CI [0.046, 0.062]), corresponding to a difference of 16.6 total crashes (30.6 vs 14.0) for an average census tract (Table A-4).

### Changes Over Time

#### Overall Trend

From 2016 to 2023, there were an average of 566 annual crashes (0.43/km), starting at 837 crashes/year (0.63 crashes/km) in 2016 and decreasing by approximately 57 crashes/year (95% CI [10, 103]), or 0.043 crashes/km each year (95% CI [−0.052, −0.033]) (Tables 1 and A-3, Figure A-1). These decreases were slightly smaller but still significant at 0.035/km/year, 95% CI [−0.059, −0.011], or 46 crashes/year when models included 2020 and 2021 fixed effects.

#### Relationship Between Streetlight Density and Time Trends

Higher streetlight density was associated with significantly greater reductions in overall crash volumes over the study period, an effect which persisted when controlling for other census-tract characteristics and 2020–2021 year fixed effects. Overall, pedestrian crashes decreased by 0.038 crashes/km each year (95% CI [0.007, 0.070]), with an additional decrease of 0.02 crashes/km/year per additional streetlight/km (95% CI [−0.003, −0.001]). This corresponded to an average decrease of 3.7 pedestrian crashes (or 57%, from 6.5 to 2.8) for census tracts at the 75th percentile streetlight density (51 streetlights/km) compared to 1.8 pedestrian crashes (or 53%, from 3.8 to 2.0) at the 25th percentile (36 streetlights/km) (Table A-5).

#### Racial Disparities

Racial disparities diminished over time, with the association between percent residents of color and pedestrian crash rates decreasing an average of 0.0038 crashes/km per 10% residents of color annually (95% CI [−0.008, −0.001]), when controlled for census tract zone, population density, and traffic volume, as well as 2020–2021 year fixed effects.

**Fig. 2:**
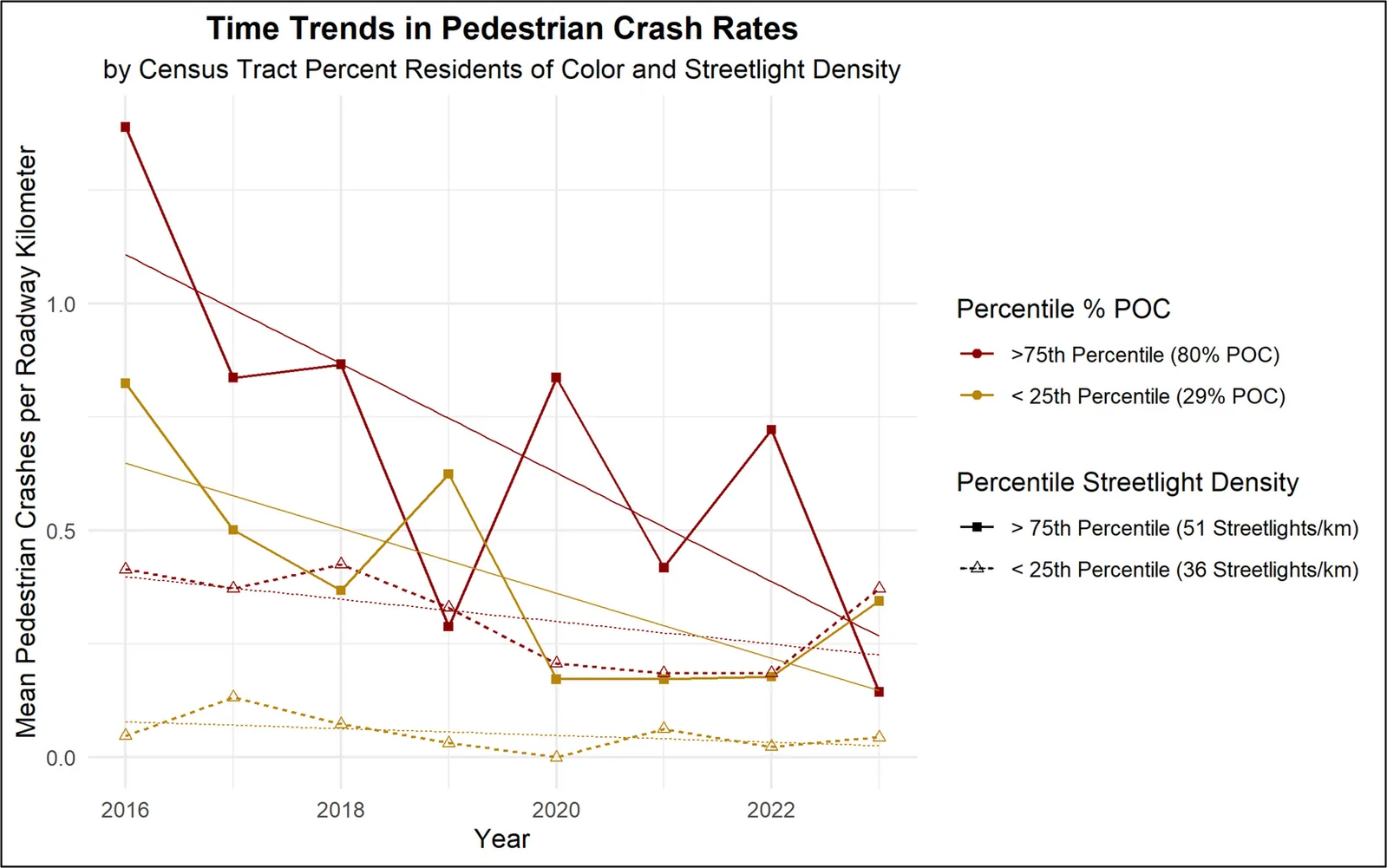
Mean annual pedestrian crashes/km over the study period, by percentile streetlight density and percentile %POC showing multiple trends as reported above: (1) crashes/km decreased overall throughout the study period; (2) census tracts with higher streetlight density (> 75th percentile) reported higher baseline pedestrian crash rates but exhibited greater reductions over time than those with lower streetlight density (represented as means for those < 25th percentile). (3) Census tracts with higher percent residents of color (> 75th percentile) experienced higher rates of pedestrian crashes, but this association also decreased over time. (4) More rapid reductions in the association between percent residents of color and crash rates were observed in census tracts with higher streetlight densities. Figure includes raw annual crash densities in crashes/km and fitted trend lines

This corresponded to a predicted average decrease of 3.1 crashes/year from 2016 to 2023 in census tracts at the 75th percentile POC (from 6.0 to 2.9), compared to a decrease of 2.4 crashes/year (from 4.0 to 1.6) at the 75th percentile, controlling for other factors. This effect was moderated by streetlight density, with the association between percent residents of color and crash density decreasing by an additional 0.0004 crashes/km/year for every additional streetlight/km in the census tract (95% CI [−0.001, −0.0002]) (Table 2; Fig. 2).

**Table 2:** Multivariable linear regression models assessing the relationship between time trends in pedestrian crash density and census tract percent residents of color, with controls for zoning category, population density, average daily traffic volume and fixed effects for pandemic years 2020 and 2021, as well as interaction terms assessing for effect moderation by streetlight density, and time trends associated with census-tract control variables

	Total annual pedestrian crashes per roadway kilometer						
	(Crashes/km/year by census tract)						
	(1)	(2)	(3)	(4)	(5)	(6)	(7)
Year	−0.043***	−0.033***	0.023*	0.031**	−0.001	−0.004	0.004
	(−0.052, −0.033)	(−0.051, −0.015)	(0.003, 0.043)	(0.011, 0.051)	(−0.042, 0.039)	(−0.046, 0.038)	(−0.038, 0.046)
10% people of color		0.047**	0.069***	0.069***	−0.130**	−0.128**	−0.128**
		(0.017, 0.077)	(0.043, 0.095)	(0.043, 0.095)	(−0.224, −0.036)	(−0.215, −0.042)	(−0.215, −0.042)
10% people of color × year		−0.002	−0.004**	−0.004**	0.013*	0.013*	0.013*
		(−0.005, 0.001)	(−0.007, −0.001)	(−0.007, −0.001)	(0.002, 0.024)	(0.003, 0.023)	(0.003, 0.023)
Downtown zoning			0.399*	0.399*		0.157	0.157
			(0.059, 0.739)	(0.059, 0.740)		(−0.191, 0.505)	(−0.191, 0.505)
Population density (1000/km^2^)			0.032***	0.032***		0.011	0.011
			(0.015, 0.048)	(0.015, 0.048)		(−0.004, 0.025)	(−0.004, 0.025)
Average daily traffic (1000 vehicles/day)			0.035**	0.035**		0.022*	0.022*
			(0.011, 0.059)	(0.011, 0.059)		(0.005, 0.040)	(0.005, 0.040)
Downtown zoning × year			−0.036	−0.036		−0.018	−0.018
			(−0.074, 0.003)	(−0.074, 0.003)		(−0.059, 0.023)	(−0.059, 0.023)
Population density (1000/km^2^) × year			−0.003**	−0.003**		−0.001	−0.001
			(−0.005, −0.001)	(−0.005, −0.001)		(−0.003, 0.0003)	(−0.003, 0.0003)
Average daily traffic (1000 vehicles/day) × year			−0.003	−0.003		−0.002	−0.002
			(−0.006, 0.0003)	(−0.006, 0.0003)		(−0.004, 0.001)	(−0.004, 0.001)
2020 year effect				−0.169***			−0.169***
				(−0.210, −0.128)			(−0.210, −0.128)
2021 year effect				−0.171***			−0.171***
				(−0.216, −0.126)			(−0.216, −0.126)
Streetlights/km					0.007	0.0003	0.0003
					(−0.003, 0.016)	(−0.011, 0.012)	(−0.011, 0.012)
Streetlights/km × year					−0.001	0.0001	0.0001
					(−0.002, 0.0004)	(−0.001, 0.001)	(−0.001, 0.001)
10% people of color × streetlights/km					0.004***	0.005***	0.005***
					(0.002, 0.007)	(0.003, 0.007)	(0.003, 0.007)
10% people of color × streetlights/km × year					−0.0004**	−0.0004***	−0.0004***
					(−0.001, −0.0001)	(−0.001, −0.0002)	(−0.001, −0.0002)
Constant	0.620***	0.368***	−0.263**	−0.257**	0.011	0.020	0.026
	(0.520, 0.720)	(0.182, 0.554)	(−0.426, −0.099)	(−0.420, −0.093)	(−0.374, 0.396)	(−0.379, 0.419)	(−0.374, 0.425)
Observations	1352	1352	1352	1352	1352	1352	1352

Linear regression model with cluster-robust standard errors at the census tract level

*p* < 0.1; *p < 0.05; **p < 0.01; ***p < 0.001

### Overnight Timing

Finally, overnight crashes (those occurring between sunset and sunrise) represented 40% of overall pedestrian crashes, with the probability of overnight occurrence increasing from 37.6% in 2016 to 45.1% in 2023, or an adjusted odds ratio (aOR) of 1.04 (95% CI = [1.02, 1.06]) per year. Census tracts with higher streetlight densities demonstrated lower overall rates of overnight crashes (aOR of 0.99; 95% CI [0.986, 0.997]), or −0.21%/streetlight/km (95% CI [−0.3%, −0.07%]) in a linear probability model, controlling for other census tract variables.

No statistically significant relationship was detected between streetlight density and time trends in relative rates of overnight crashes. Overall, higher streetlight densities were associated with more rapid decreases in both daytime and overnight crashes, by 0.0005 crashes/km/year per streetlight/km from dusk to dawn (95% CI [−0.001, −0.0002]) and 0.0014 (95% CI [−0.002, −0.001]) crashes/km/year per streetlight/km during daylight hours (Table A-6).

Sensitivity analyses using 2016 data were performed with no meaningful differences in main findings.

## Discussion

Overall rates of pedestrian-motor vehicle crashes in Boston decreased markedly over the 2016–2023 interval, and census tracts with higher streetlight densities experienced greater reductions during both daytime and overnight hours. Census tracts with larger minority populations experienced persistently higher pedestrian crash rates. However, these disparities decreased over time, and higher streetlight density was associated with greater reductions in race-related differences.

Multiple potential mechanisms (or combinations thereof) could underlie these associations. One possibility is that they represent a direct effect of roadway lighting. Streetlights commonly activate not only overnight but also during low-light or low-visibility conditions (e.g., heavy precipitation, dense fog, or other inclement weather) that also impact roadway safety and pedestrian risk [25, 27–29]. Thus, improved low-light visibility may lead to fewer overnight and daytime crashes if daytime crashes more often occur during periods of reduced visibility.

Alternatively, streetlight density may serve as a proxy measure for investment in local roadways or pedestrian safety infrastructure (including sidewalks, crosswalks, pedestrian signals, etc.) that we did not observe in our data. Streetlight density could also be related to additional unobserved roadway or neighborhood features shaping traffic patterns or speeds (such as lane widths or intervals between stops). Any (or all) of these could also contribute to lower pedestrian crash rates [26, 30].

More indirectly, census tracts with higher streetlight densities could be experiencing broader changes in demographic factors (such as median age or income) or local commercial activity (and thereby foot traffic) that impact pedestrian volumes, behaviors, or vulnerability.

With respect to racial disparities, any of these mechanisms (or others) could also underly the association between streetlight density and decreasing racial disparities. Especially, given previous work documenting the insufficiency of pedestrian safety infrastructure in historically-minority neighborhoods, streetlight density could represent a direct or proxy measure for infrastructural investments that reduce excess risk [6].

Further, the share of pedestrian crashes occurring overnight increased from 2016 to 2023. Higher streetlight density was associated with lower relative rates of overnight crashes, but not lower year-to-year increases in this proportion. This finding is consistent with results from prior work suggesting that increases in the proportion of overnight crashes may be driven by population movement or other factors with limited sensitivity to roadway lighting [2].

This study has several key limitations. First, it represents a retrospective analysis of a single city, with unknown generalizability to other contexts. Second, it uses streetlight, demographic, and roadway data from single time points, and changes in these variables over the study period could not be assessed. Third, our data include the 2020–2021 interval, during which there were significant aberrations in commuting and travel behavior related to the COVID-19 pandemic [23]. We did include modeling elements to control for this. However, there may have been volatility, especially in the day/night distribution of crashes, that made trends more difficult to detect. Finally, this work is descriptive in design and cannot establish causal mechanisms.

Overall, our analysis found that urban streetlight density was associated with decreasing pedestrian crash rates and decreasing racial disparities in crash incidence. Further work is needed to better understand the mechanisms underlying these associations in order to help develop targeted interventions that directly improve pedestrian safety.

## Supplementary Information

Below is the link to the electronic supplementary material.ESM 1(PDF 990 KB)

## Data Availability

All data sources are public and linked in the references.
