# Supplementary material for: The Impact of Streetlight Placement on Trends and Disparities in Pedestrian-Motor Vehicle Crashes
Source: J Urban Health. 2026 Jun 30;103(4):791–801. doi: 10.1007/s11524-026-01098-8 (PMC13525136; doi:10.1007/s11524-026-01098-8)
Supplement: Supplementary file 1 [file 11524_2026_1098_MOESM1_ESM.pdf]

Online Appendix:

Supplementary Tables and Figures for

The Impact of Streetlight Placement on Trends and Disparities in Pedestrian-Motor Vehicle Collisions

Census Tract Characteristics Associated with Streetlight Density

	Streetlights per Roadway Kilometer (by Census Tract)					
	Streetlights/km/year (2016 Inventory)			Streetlights/km/year (2022 Inventory)		
	(1)	(2)	(3)	(4)	(5)	(6)
Total Pedestrian Crash Density in Preceding Years (Crashes/km/year)	21.47***		10.53***	22.44***		13.85***
	(16.75, 26.20)		(6.25, 14.81)	(15.29, 29.59)		(8.02, 19.68)
Downtown Zoning		22.77***	19.60***		21.66***	20.75***
		(14.67, 30.86)	(10.98, 28.22)		(12.89, 30.43)	(13.25, 28.25)
Population Density (1000/km^2)		1.99***	1.77***		1.06***	0.76***
		(1.46, 2.52)	(1.27, 2.27)		(0.61, 1.51)	(0.33, 1.19)
Average Daily Traffic (1000 Vehicles/Day)		0.96**	0.66*		0.43	0.03
		(0.30, 1.62)	(0.03, 1.29)		(-0.25, 1.11)	(-0.55, 0.61)
Constant	23.32***	10.72***	8.71**	33.21***	29.15***	27.75***
	(18.66, 27.98)	(4.88, 16.56)	(3.39, 14.03)	(30.24, 36.18)	(25.53, 32.78)	(24.66, 30.85)
Observations	169	169	169	169	169	169

Note: .p < 0.1; *p < 0.05; **p < 0.01; *** p < 0.001

Linear regression model with cluster-robust standard errors at the census tract level

Table A-1 Multivariable linear regression models assessing the relationship between streetlight density and zoning category, population density, and average daily traffic volume, as well as pedestrian crash densities in the preceding years (2015 for the 2016 inventory, 2016-2021 for the 2022 inventory)

Racial Disparities in Streetlight Density

	Streetlights per Roadway Kilometer (by Census Tract)			
	Streetlights/km/year (2016 Inventory)		Streetlights/km/year (2022 Inventory)	
	(1)	(2)	(3)	(4)
Ten Percent Residents of Color	-0.67	-1.72^{**}	-0.62	-1.63^{***}
	(-2.08, 0.75)	(-2.85, -0.58)	(-1.53, 0.28)	(-2.38, -0.88)
Total Pedestrian Crash Density in Preceding Years (Crashes/km/year)		23.45^{***}		25.58^{***}
		(18.21, 28.70)		(18.57, 32.59)
Constant	39.05^{***}	31.39^{***}	46.60^{***}	40.55^{***}
	(29.42, 48.68)	(23.36, 39.42)	(39.74, 53.47)	(35.20, 45.90)
Observations	169	169	169	169
Note:				**.**p < 0.1; *p < 0.05; **p < 0.01; *** p < 0.001
				Linear regression model with cluster-robust standard errors at the census tract level

Table A-2 Multivariable linear regression models assessing the relationship between streetlight density and census tract residents of color, with secondary models controlling for pedestrian crash densities in the preceding years (2015 for the 2016 inventory, 2016-2021 for the 2022 inventory)

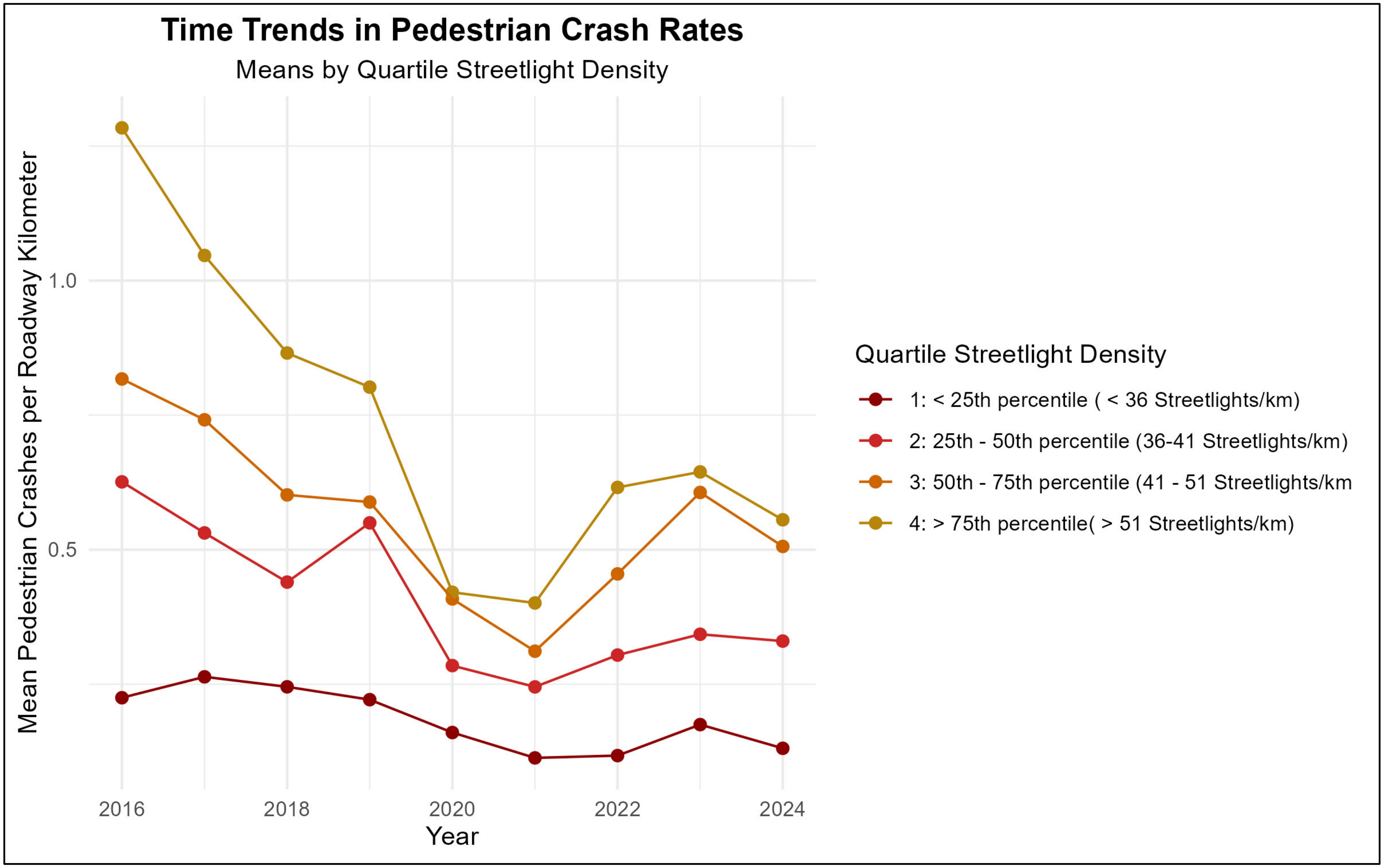

Fig. A-1 Mean annual pedestrian crashes/km over the study period, by percentile streetlight density. Figure shows persistent differences in mean crash densities, as well as decreases over time.

Overall Trend and Census Tract Characteristics Associated with Overall Pedestrian Crash Rates

	Total Annual Pedestrian Crashes per Roadway Kilometer (by Census Tract)						
	N. Crashes	Crashes/km/year					
	(1)	(2)	(3)	(4)	(5)	(6)	(7)
Year	-56.679*	-0.043*	-0.035*				
	(-103.284, -10.073)	(-0.078, -0.008)	(-0.059, -0.011)				
2020 Year Effect			-0.169*				
			(-0.332, -0.006)				
2021 Year Effect			-0.171*				
			(-0.339, -0.003)				
Downtown Zoning				0.294**			0.063
				(0.105, 0.483)			(-0.148, 0.273)
Population Densit (1000/km^2)					0.024***		0.020**
					(0.012, 0.036)		(0.008, 0.032)
Average Daily Traffic (1000 Vehicles/Day)						0.034**	0.028**
						(0.013, 0.055)	(0.010, 0.046)
Constant	821.179***	0.620***	0.626***	0.383***	0.238***	0.232***	0.103.
	(585.832, 1,056.525)	(0.442, 0.798)	(0.509, 0.743)	(0.315, 0.452)	(0.121, 0.354)	(0.123, 0.341)	(-0.001, 0.206)
Observations	8	8	8	169	169	169	169
Note:	.p < 0.1; *p < 0.05; **p < 0.01; *** p < 0.001						
	Linear regression model with cluster-robust standard errors at the census tract level						

Table A-3 Multivariable linear regression models assessing the relationship between pedestrian crash counts or densities (crashes/km/year) and census tract characteristics including streetlight density, zoning category, population density, and average daily traffic volume

Relationship Between Racial Composition and Pedestrian Crashes

	Total Annual Pedestrian Crashes per Roadway Kilometer		
	(Crashes/km/year by Census Tract)		
	(1)	(2)	(3)
Ten Percent People of Color	0.039^{***}	0.052^{***}	0.047^{***}
	(0.018, 0.059)	(0.034, 0.070)	(0.030, 0.065)
Downtown Zoning		0.239^{*}	-0.068
		(0.043, 0.435)	(-0.405, 0.269)
Population Density (1000/km^2)		0.018^{**}	0.017^{**}
		(0.007, 0.028)	(0.007, 0.027)
Average Daily Traffic (1000 Vehicles/ Day)		0.023^{**}	0.024^{***}
		(0.008, 0.039)	(0.010, 0.039)
Ten Percent People of Color x Downtown Zoning			0.097^{*}
			(0.009, 0.184)
Constant	0.220^{***}	-0.159^{**}	-0.134^{*}
	(0.103, 0.337)	(-0.264, -0.055)	(-0.236, -0.032)
Observations	169	169	169
Note:	.p < 0.1; *p < 0.05; **p < 0.01; *** p < 0.001		
	Linear regression model with cluster-robust standard errors at the census tract level		

Table A-4 Multivariable linear regression models assessing the relationship between pedestrian crash density and census tract percent residents of color, with controls for census tract characteristics including streetlight density, zoning category, population density, and average daily traffic volume, as well as an interaction showing differences in the relationship between percent residents of color and pedestrian crash density by zoning category

Relationship Between Streetlight Density and Time Trends in Pedestrian Crashes

	Total Annual Pedestrian Crashes per Roadway Kilometer (by Census Tract)				
	Crashes/km/year				
	(1)	(2)	(3)	(4)	(5)
Year	-0.043***	0.038*	0.038*	0.048*	0.056**
	(-0.052, -0.033)	(0.007, 0.070)	(0.007, 0.070)	(0.010, 0.086)	(0.018, 0.094)
Streetlights/km		0.022***	0.022***	0.020***	0.020***
		(0.014, 0.029)	(0.012, 0.031)	(0.009, 0.031)	(0.009, 0.031)
Streetlights/km x Year		-0.002***	-0.002***	-0.002*	-0.002*
		(-0.003, -0.001)	(-0.003, -0.001)	(-0.003, -0.0003)	(-0.003, -0.0003)
Downtown Zoning			-0.221*	-0.266	-0.266
			(-0.439, -0.002)	(-0.617, 0.084)	(-0.617, 0.084)
Population Density (1000/km^2)			0.006	0.013	0.013
			(-0.005, 0.017)	(-0.003, 0.030)	(-0.003, 0.030)
Average Daily Traffic (1000 Vehicles/Day)			0.022**	0.033***	0.033***
			(0.009, 0.036)	(0.014, 0.052)	(0.014, 0.052)
Downtown Zoning x Year				0.010	0.010
				(-0.029, 0.049)	(-0.029, 0.049)
Population Density (1000/km^2) x Year				-0.002.	-0.002.
				(-0.004, 0.0002)	(-0.004, 0.0002)
Average Daily Traffic (1000 Vehicles/Day) x Year				-0.002.	-0.002.
				(-0.005, 0.0001)	(-0.005, 0.0001)
2020 Year Effect					-0.169***
					(-0.210, -0.128)
2021 Year Effect					-0.171***
					(-0.216, -0.126)
Constant	0.620***	-0.311*	-0.451**	-0.493**	-0.488**
	(0.520, 0.720)	(-0.621, -0.001)	(-0.779, -0.124)	(-0.852, -0.135)	(-0.846, -0.129)
Observations	1,352	1,352	1,352	1,352	1,352
Note:	.p < 0.1; *p < 0.05; **p < 0.01; *** p < 0.001				
	Linear regression model with cluster-robust standard errors at the census tract level				

Table A-5 Multivariable linear regression models assessing the relationship between time trend in pedestrian crash density and census tract streetlight density, with controls for zoning category, population density, and average daily traffic volume, as well as time trends associated with these variables, and fixed effects for pandemic years 2020 and 2021

Relationship Between Overnight Occurance and Streetlight Density

	Probability of Occuring Overnight								
	Percent Chance (Linear Probability Model)		Adjusted Odds Ratio (Logistic Regression Model)					Daytime Crashes/km	Overnight Crashes/km
	OLS		*logistic*					*OLS*	*OLS*
	(1)	(2)	(3)	(4)	(5)	(6)	(7)	(8)	(9)
Year	0.888***	0.982***	1.037***		1.038***	1.041***	1.022	0.029**	0.010
	(0.444, 1.333)	(0.537, 1.428)	(1.019, 1.057)		(1.019, 1.056)	(1.023, 1.061)	(0.967, 1.081)	(0.008, 0.050)	(-0.004, 0.023)
Streetlights/km		-0.207**		0.996.	0.991**	0.991**	0.990**	0.014***	0.007***
		(-0.341, -0.073)		(0.992, 1.001)	(0.986, 0.997)	(0.986, 0.997)	(0.982, 0.997)	(0.009, 0.020)	(0.004, 0.010)
Downtown Zoning		6.110.			1.297*	1.292.	1.291.		
		(-0.065, 12.284)			(1.000, 1.682)	(0.995, 1.678)	(0.995, 1.676)		
Population Density (1000/km^2)		-0.031			0.999	0.999	0.999		
		(-0.338, 0.275)			(0.986, 1.012)	(0.986, 1.012)	(0.986, 1.012)		
Average Daily Traffic (1000 Vehicles/Day)		0.360.			1.015.	1.015.	1.015.		
		(-0.031, 0.751)			(0.999, 1.032)	(0.999, 1.031)	(0.999, 1.032)		
2020 Year Effect		-1.430				0.943	0.943		
		(-6.520, 3.661)				(0.763, 1.166)	(0.763, 1.165)		
2021 Year Effect		-5.785*				0.785*	0.786*		
		(-11.282, -0.287)				(0.620, 0.994)	(0.621, 0.994)		
Streetlights/km x Year							1.000	-0.001***	-0.001**
							(0.999, 1.001)	(-0.002, -0.001)	(-0.001, -0.0002)
Constant	37.004***	44.007***	0.589***	0.848	0.786	0.790	0.858	-0.234*	-0.077
	(34.377, 39.632)	(37.110, 50.905)	(0.527, 0.658)	(0.662, 1.088)	(0.589, 1.048)	(0.590, 1.058)	(0.594, 1.239)	(-0.441, -0.028)	(-0.197, 0.043)
Observations	4,956	4,956	4,956	4,956	4,956	4,956	4,956	1,352	1,352

Note: .p < 0.1; *p < 0.05; **p < 0.01; *** p < 0.001

Linear regression model with cluster-robust standard errors at the census tract level

Table A-6 Multivariable linear and logistic regression models assessing the relationship between overnight crash occurrence and census tract characteristics, including zoning category, population density, average daily traffic volume, with fixed effects for pandemic years 2020 and 2021, as well as interaction terms assessing for effect moderation by streetlight density

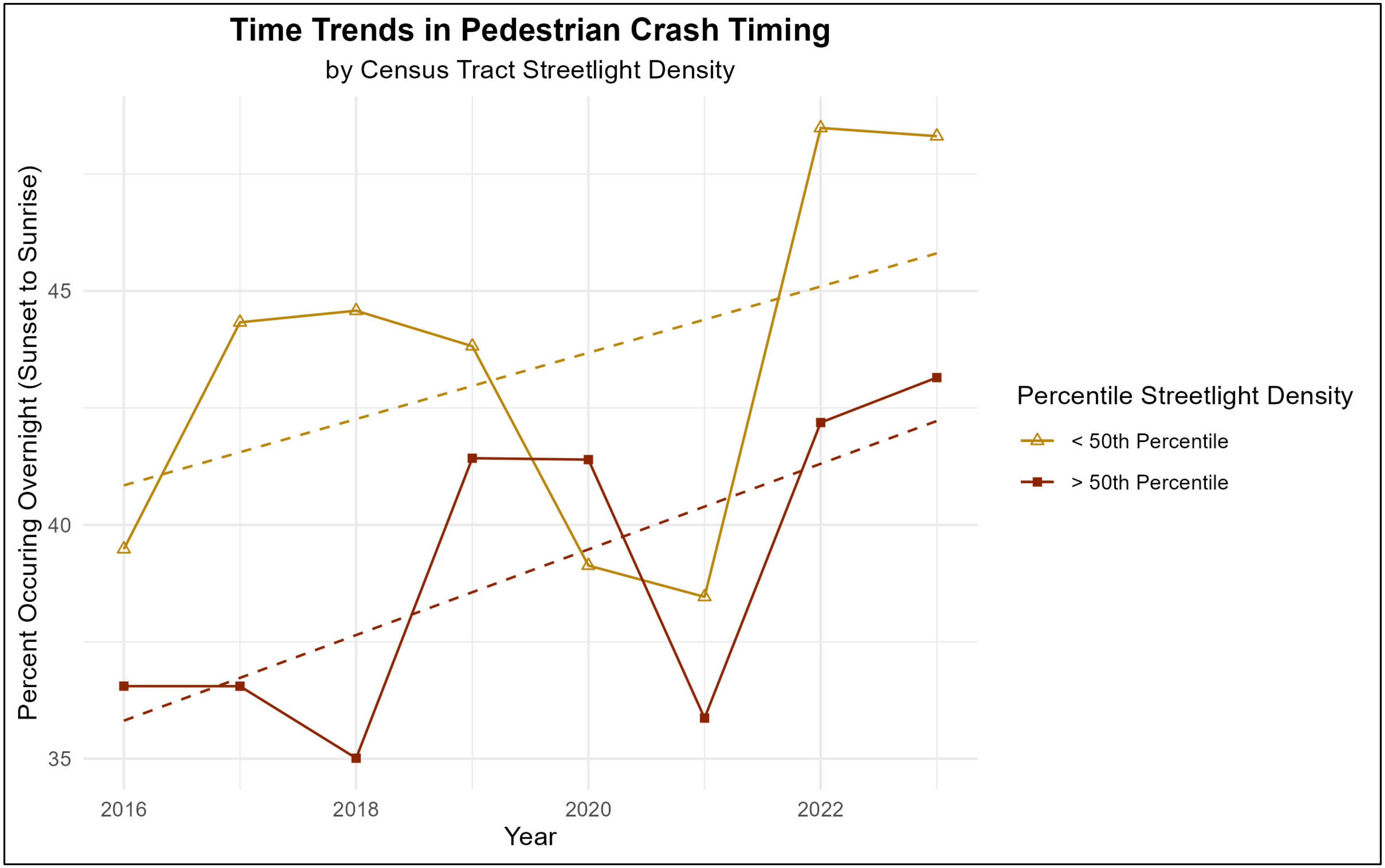

Fig. A-2 Mean annual percent pedestrian crashes occurring overnight (EMS dispatch time between sunset and sunrise) over the study period, by percentile streetlight density. Raw data and regression lines demonstrate both an overall increase in overnight proportion and lower rates of overnight crashes in census tracts with high (≥75th %ile) streetlight density (streetlights/km)
